# Case Report: Prenatal imaging and genetic integrated diagnosis of *SCN2A* encephalopathy—a case of cryptical cortical dysplasia caused by a loss-of-function frameshift genetic variant

**DOI:** 10.3389/fnins.2026.1698102

**Published:** 2026-02-04

**Authors:** Linyan Zhu, Mei Chen, Huiqing Ding, Minyue Dong

**Affiliations:** 1 Women’s Hospital, School of Medicine, Zhejiang University, Hangzhou, China; 2 Department of Obstetrics and Gynaecology, The First Affiliated Hospital of Ningbo University, Ningbo, China; 3 Key Laboratory of Reproductive Genetics, Ministry of Education, Zhejiang University, Hangzhou, China

**Keywords:** cortical dysplasia, frameshift mutation, genotype–phenotype, prenatal diagnosis, *SCN2A*

## Abstract

**Objective:**

*SCN2A* mutations are linked to postnatal epileptic encephalopathies, but prenatal features are poorly defined. We describe a novel *SCN2A* frameshift mutations prenatal phenotype and genotype–phenotype correlations.

**Case report:**

A fetus with progressive cerebral anomalies underwent serial ultrasound, MRI, and whole-exome sequencing. Imaging showed persistent cavum septi pellucidi narrowing (0.9–2.6 mm at 21–30 weeks) and focal cortical thickening at the left temporoparietal junction. A *de novo* heterozygous *SCN2A* frameshift mutation (c.3043del, p.D1015Lfs*22) was identified, truncating Nav1.2 at residue 1015 and ablating critical functional domains. Protein modeling confirmed complete loss-of-function (LoF) due to α-subunit disruption.

**Conclusion:**

This is the first report of prenatal imaging phenotypes in *SCN2A* frameshift mutations, featuring persistent CSP narrowing and progressive focal cortical thickening. Distinct from missense mutation-related ventriculomegaly, it suggests potential mutation-specific signatures. Unexplained CSP narrowing/cortical thickening warrants sodium channelopathy suspicion, with *SCN2A* prioritized in fetal cortical malformation panels. Single-case limitations demand large-cohort validation for genotype–phenotype correlations.

## 1 Introduction

The *SCN2A* gene encodes the voltage-gated sodium channel Nav1.2, with mutations linked to a broad spectrum of epileptic encephalopathies, ranging from benign infantile seizures to lethal neonatal forms (Rusina et al., 2024). Pathogenic variants in the *SCN2A* are linked to childhood-onset epilepsy of varying severity, autism spectrum disorder (ASD) with or without seizures, and nonsyndromic intellectual disability (ID) (Thompson et al., 2023). Previous research suggests that the primary phenotypes of *SCN2A*-related disorders (ranging from neonatal-, infant- and later-onset epilepsy to autism without seizures) are strongly correlated with Nav1.2 channel variant function, with gain-of-function or mixed variants predominating in neonatal-onset epilepsy, moderate loss-of-function variants in infant-onset epilepsy, and severe/complete loss-of-function variants in later-onset epilepsy and autism, while non-seizure severity is jointly determined by seizure onset age and variant function (Berg et al., 2024). However, this conclusion is based on limited and varied studies, and most disease-related *SCN2A* variants remain uncharacterized (Thompson et al., 2023). Mutations in the *SCN2A* gene have been identified in newborns and adults across diverse ethnicities (Clatot et al., 2025). However, only two cases describing the prenatal imaging features in fetuses have been reported, both of which involve missense mutations in *SCN2A*. One study identified the earliest severe prenatal form of early-onset epileptic encephalopathies (EOEE) due to *SCN2A* missense mutation with a 30-week ultrasound showing severe bilateral ventriculomegaly (Sauvestre et al., 2017). Another report found severe ventriculomegaly and cortical malformations in a 32-week fetus with an *SCN2A* missense mutation (Bernardo et al., 2017). To date, no reports exist on the prenatal imaging characteristics of frameshift mutations in *SCN2A* gene. While postnatal symptoms of *SCN2A*-related disorders, especially those with cortical malformation, are well-known, prenatal imaging features remain unclear despite the growing recognition of sodium channelopathies’ impact on neurodevelopment before birth. Given the severe postnatal outcomes, prenatal diagnosis is crucial when possible.

Current prenatal ultrasounds focus on major structural anomalies and are not sensitive enough for detecting subtle cortical issues. Although advanced fetal MRI can detect small cortical irregularities, it is seldom used for isolated CSP narrowing or asymmetrical sulcation (Fantasia et al., 2023). *SCN2A*-related fetal anomalies often resemble those caused by chromosomal disorders or intrauterine infections, leading to misdiagnoses and missed genetic causes (Schwarz et al., 2016).

Few studies have explored the relationship between fetal CSP size and development. A cross-sectional fetal study found that 31.2% of otherwise healthy newborns, prenatally diagnosed with an isolated wide or narrow cavum septi pellucidi (CSP), screened positive for developmental delay and behavioral abnormalities, yet its findings indicated that such CSP variations do not increase the risk of suspected developmental or behavioral milestone delays (Sichitiu et al., 2024). We present the first in utero imaging of a fetus with a prenatal *SCN2A* frameshift mutation, showing persistent CSP narrowing and focal cortical thickening. This sheds light on the developmental impact of *SCN2A* mutations and underscores the significance of genotype–phenotype correlations in prenatal diagnosis.

## 2 Materials and methods

### 2.1 Case report

#### 2.1.1 Maternal history

A 28-year-old primigravida (G1P0) with a non-consanguineous marriage and no family history of genetic disorders received routine prenatal care. The pregnancy was spontaneously conceived, with no known teratogen exposure.

#### 2.1.2 Ultrasound findings

**Figure 1:**
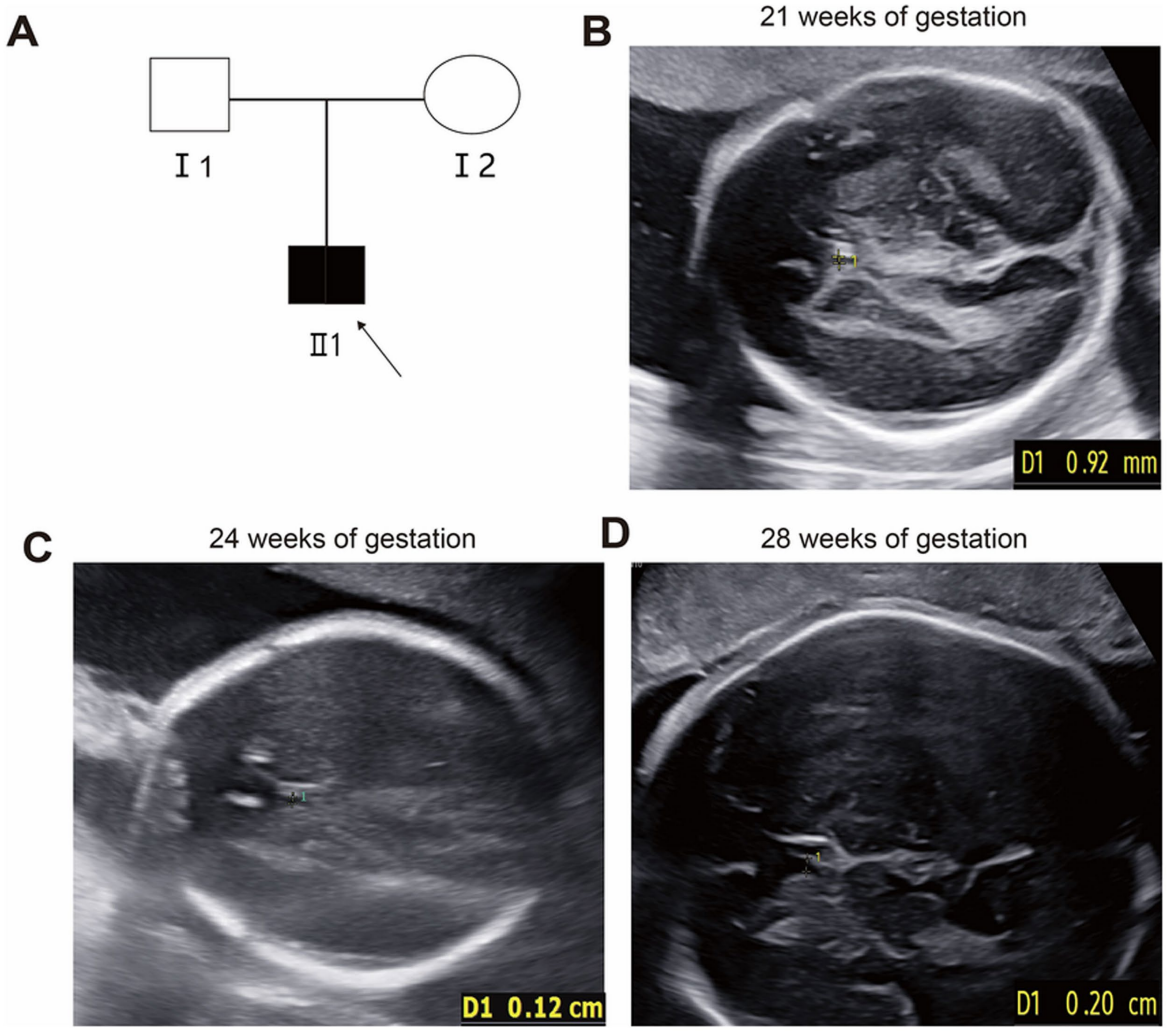
Pedigree and fetal ultrasound scan findings. **(A)** Pedigree of the family. The solid square (male) represented the affected fetus. **(B–D)** Fetal ultrasound indicated persistent narrowing of the cavum septi pellucidi (CSP). The specific measurements were 0.9 mm at 21 weeks of gestation **(B)**, 0.12 cm at 24 weeks of gestation **(C)**, and 0.20 cm at 28 weeks of gestation **(D)**.

At 21 weeks of gestation, fetal ultrasound demonstrated a cavum septi pellucidi (CSP) width of 0.9 mm (Figure 1B), below the normal lower limit of 3 mm. Subsequent ultrasounds at 24 and 28 weeks showed progressive widening of the CSP to 1.2 mm and 2.0 mm, respectively (Figures 1C,D).

#### 2.1.3 MRI findings

**Figure 2:**
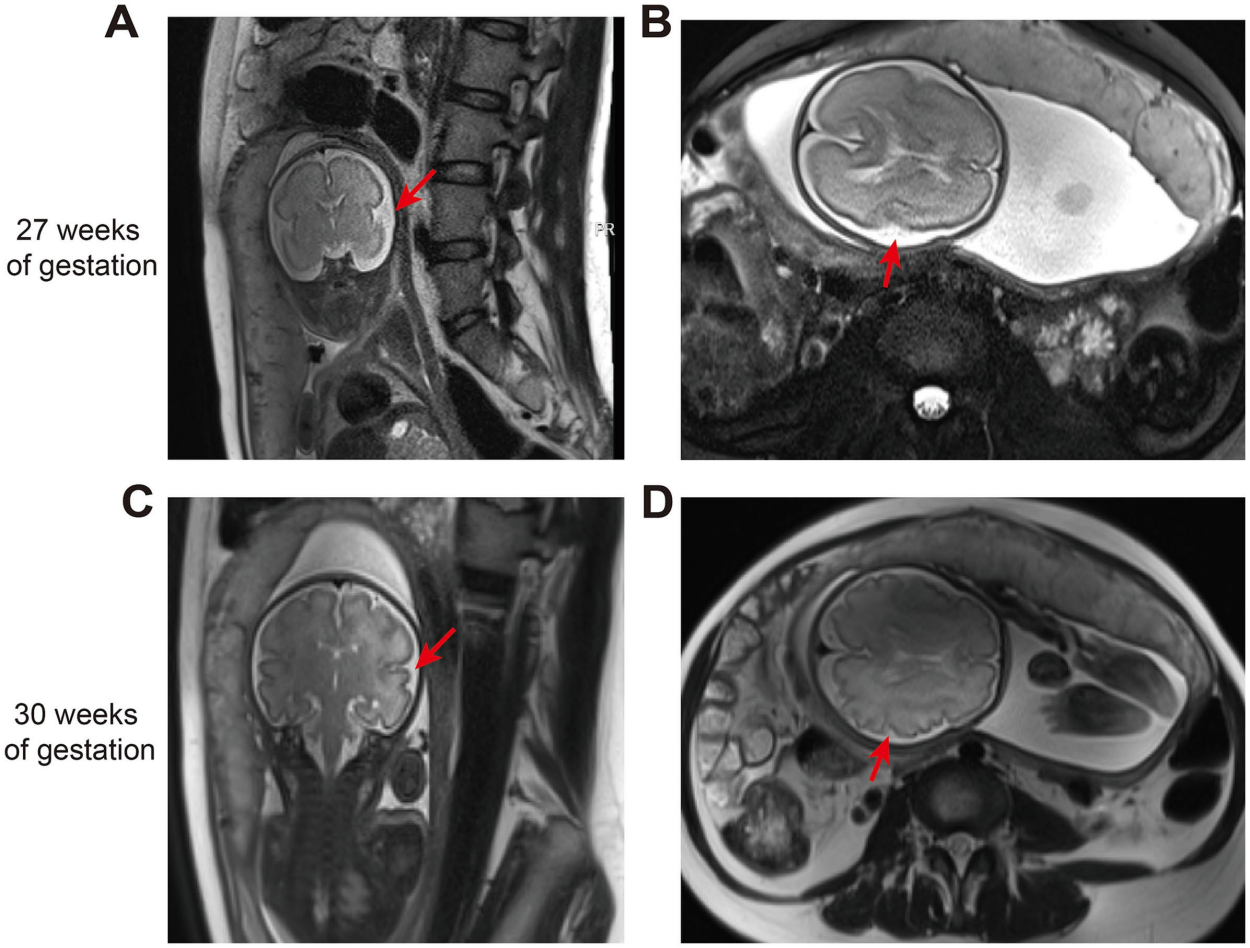
The fetal MRI findings. **(A,B)** The fetal brain MRI showed focal cortical thickening and sulcal deepening at the left temporoparietal junction at 27 weeks of gestation. **(C,D)** The fetal brain MRI showed progressive sulcal deepening and cortical thickening at the left temporoparietal region, with adjacent white matter hyperintensity.

Fetal MRI performed at 27 weeks revealed a CSP width of 2.6 mm, along with focal cortical thickening and sulcal deepening at the left temporoparietal junction (Figures 2A,B). A repeat MRI at 30 weeks showed progression of sulcal deepening and cortical thickening in the same region, accompanied by adjacent white matter hyperintensity (Figures 2C,D). Key imaging features are summarized in Table 1.

**Table 1:** Imaging findings of ultrasound and MRI at different gestational weeks.

GW	US (CSP: mm)	MRI
21	0.9	NA
24	1.2	NA
27	NA	Focal cortical thickening and sulcal deepening at the left temporoparietal junction. CSP 2.5 mm
28	2.0	NA
30	NA	Progressive sulcal deepening and cortical thickening at the left temporoparietal region, with adjacent white matter hyperintensity. CSP 2.6 mm

GW, gestational week; US, ultrasound; MRI, magnetic resonance imaging.

#### 2.1.4 Genetic findings

**Figure 3:**
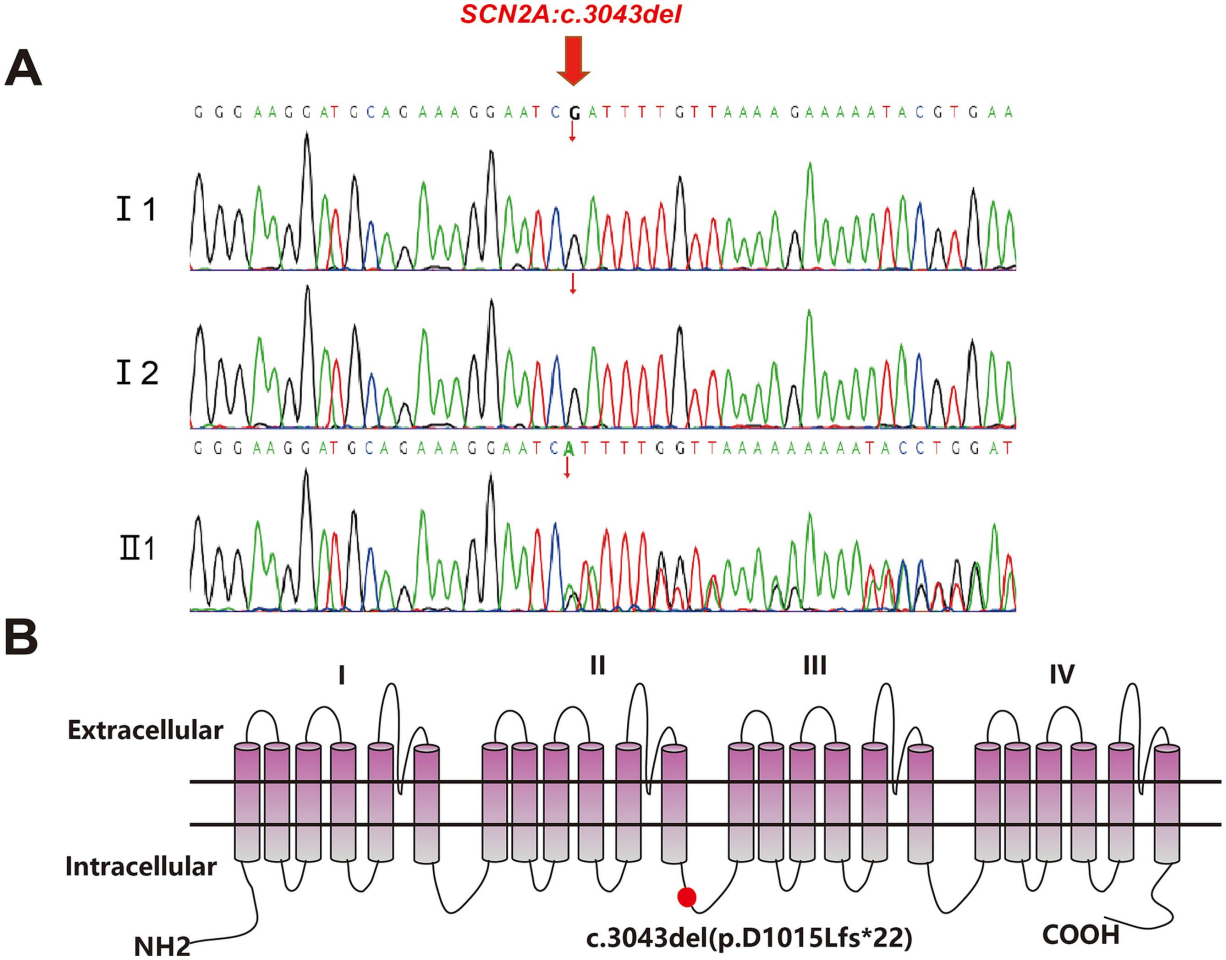
Sanger sequencing analysis and the topology diagram of the human Nav1.2 channel’s α subunit. **(A)** The variant c.3043del was validated by Sanger sequencing (red arrows indicated the mutation). I1 and I2: wildtype genotype of the fetus’s parents. II1: mutation genotype of the fetus c.3043del (p.D1015Lfs*22). **(B)** Topology diagram of the human Nav1.2 channel’s α subunit. The location of the D1015Lfs*22 variant described in the study is shown by a red circle.

Amniocentesis was performed at 29 weeks. Karyotyping and chromosomal microarray identified no abnormalities. Whole-exome sequencing, however, detected a heterozygous frameshift mutation in the *SCN2A* gene: c.3043del (p.D1015Lfs*22). Parental testing confirmed the mutation was *de novo* (Figure 3A). This variant is predicted to cause premature termination of the Nav1.2 protein at residue 1015, resulting in loss of the III–IV domains and the C-terminal region (Figure 3B).

#### 2.1.5 Outcome

A multidisciplinary team was consulted, and the variant was classified as pathogenic, associated with developmental and epileptic encephalopathy 11 (DEE11). Following counseling, the parents elected to terminate the pregnancy. A male fetus weighing 1800 g was delivered, with no obvious dysmorphic features. Postmortem examination was declined by the parents.

This study was approved by the Ethics Committee of The First Affiliated Hospital of Ningbo University and conformed to the Declaration of Helsinki. All participants provided their written informed consents.

### 2.2 WES and data analysis

WES was conducted as previously described (Zhu et al., 2024). Genetic variant screening was conducted against pathogenic variant databases, normal genomic reference datasets, clinical datasets of 2,000 genetic disorders, and advanced computational algorithms for variant prioritization. To identify potential genetic etiologies, whole-exome sequencing (WES) was conducted on the proband (fetus) and their parental samples. The genome build version hg19 was employed for sequence alignment and variant annotation. For the fetal proband, sequencing yielded 12.5G of data, with a Q30 quality value of 94.67% (indicating high base-calling accuracy). Coverage metrics showed a 20X coverage rate of 99.41% (reflecting robust coverage of the target exomic regions) and an average sequencing depth of 171.79X, which meets the standard requirements for reliable variant detection in clinical genetic analyses. Parental WES data (included for trio analysis) were also generated with consistent quality: 13.7G of data (Q30: 94.98%, 20X coverage: 99.44%, average depth: 187.38X) for the father, and 12.1G of data (Q30: 94.29%, 20X coverage: 99.13%, average depth: 166.50X) for the mother.

### 2.3 Sanger sequencing

Sanger sequencing was carried out to validate the variants. The primers were as follows: forward: 5′-GCCTTGCTTTTGAGTTCCTTC-3′ and reverse:5′-TAGTAGTTCCATTTCCGTCTTTGA′ for *SCN2A* with the PCR protocol template: 95 °C, 10 min; then 35 cycles of 94 °C, 30 s, 60 °C, 30 s, and 72 °C, 30 s; then 72 °C, 10 min. The products were sequenced with the ABI 3730 DNA analyzer (Applied Biosystems).

### 2.4 Bioinformatics analysis

Web online software SWISS-MODEL^1^ was used to make three-dimensional protein structure models. PyMOL 2.5 Viewer software was used to visualize and analyze the impact of the variant on the protein structure.

## 3 Results

### 3.1 Genomic analysis and protein structure prediction

**Figure 4:**
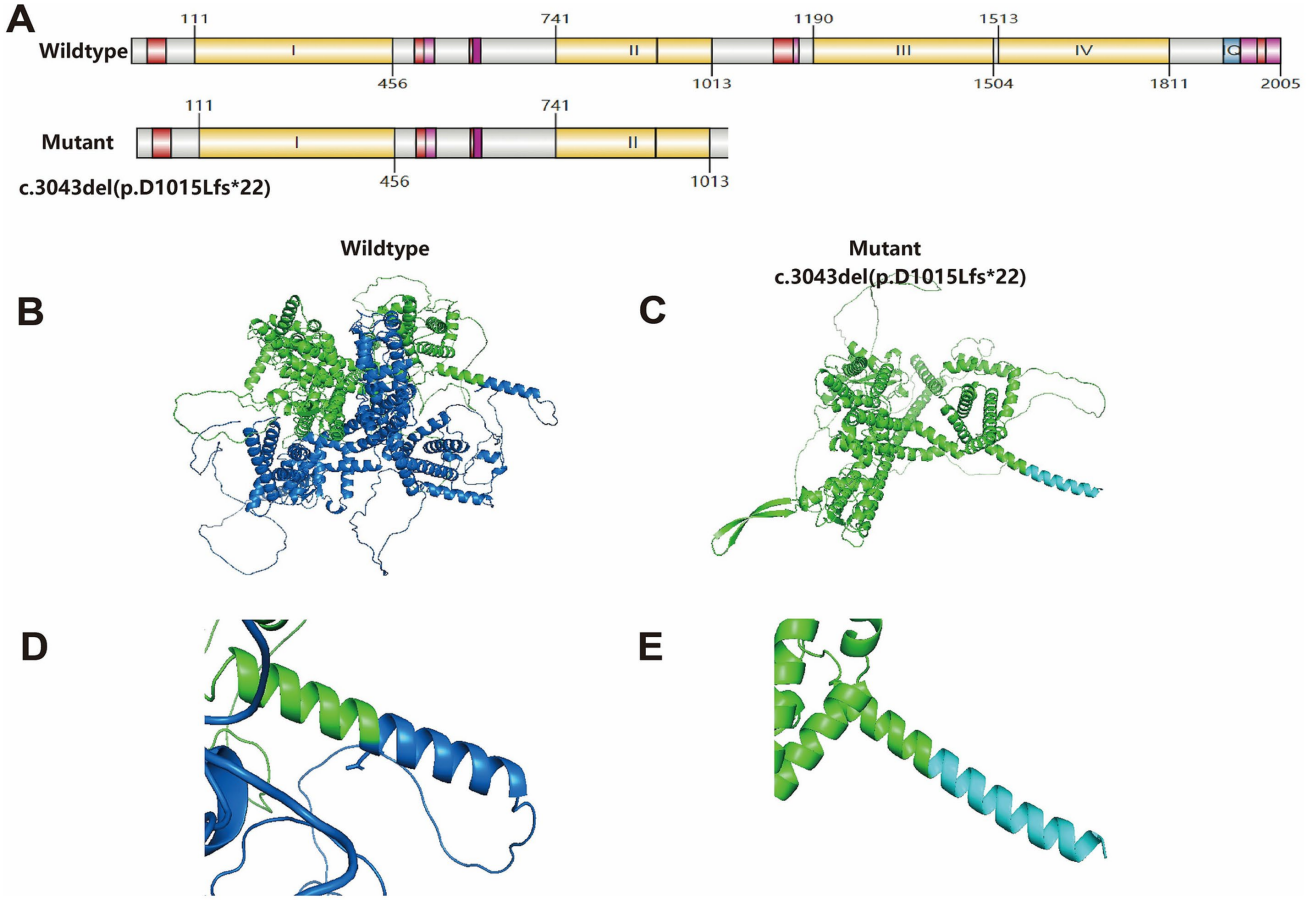
Structure of the *SCN2A* wild-type and c.3043del (p.D1015Lfs*22) mutation protein. **(A)** Schematic depicting wildtype *SCN2A* (UniProt Q99250) and the truncated mutant protein domain arrangement and boundaries. Generated using IBS 2.0103. **(B)** Overview of the three-dimensional structure of the *SCN2A* wild-type protein. **(C)** Overview of the three-dimensional structure of the *SCN2A* D1015Lfs*22 variant protein. **(D)** Details structure of the *SCN2A* wild-type protein around the mutated site. **(E)** Details of the *SCN2A* D1015Lfs*22 variant protein around the mutated site. The structures of **(B–E)** were all predicted by the PyMOL 2.5 Viewer software.

WES revealed a novel frameshift variant (c.3043del) in exon 17 of the *SCN2A* gene in the fetus. The c.3043del mutation creates a premature termination codon, resulting in truncation of the encoded sodium channel protein. Protein structure of c.3043del mutation starting from amino acid position 1015 was changed (Figure 4A) (Xie et al., 2022), and the three-dimensional structure was predicted on the SWISS-MODEL workspace and analyzed by PyMOL software^2^ (Figures 4B–E). This aberrant truncation eliminates critical III-IVdomains and C-terminal domain, ultimately leading to complete loss-of-function (LOF) of the ion channel due to structural disruption of the pore-forming α-subunit.

### 3.2 Literature review

To better understand the clinical manifestations of fetus with *SCN2A* mutations. The data from previously reported cases were collected from the literature. Only two reports in the literature have described the prenatal imaging phenotypes in two fetuses with *SCN2A* gene mutations. The clinical and laboratory parameters are shown in Table 2.

**Table 2:** The genetic testing results and prenatal clinical features of 3 fetuses with *SCN2A* variants.

Patient variation	Inheritance	GW	MRI	US	Outcome	PMID
c.751G>A, (p.Val251Ile)	*de novo*	32w	Severe ventriculomegaly Pachygyria/abnormal cortical gyration Thin corpus callosum	Bilateral ventriculomegaly	Live birth frequent epilepsy	28254201
c.4471A>G, (p.Thr1491Ala)	*de novo*	30w	None	Severe symmetrical bilateral ventriculomegaly, distal arthrogryposis	TOP at 32 weeks	28489313
c.3043del (p.D1015Lfs*22)	*de novo*	32w	Cortical thickening abnormal cortical gyration narrow cavum septi pellucidi (CSP)	Narrow cavum septi pellucidi (CSP)	TOP at 34 weeks	Our study

Both prior cases harbored *de novo SCN2A* mutations, diagnosed at 30 and 32 weeks of gestation. Ventriculomegaly was the core ultrasonic abnormality in both: Case 1 (32 weeks) showed severe ventriculomegaly, pachygyria/abnormal cortical gyration, and thin corpus callosum on MRI, with live birth followed by frequent epileptic seizures; Case 2 (30 weeks) had severe symmetrical bilateral ventriculomegaly combined with distal arthrogryposis, no MRI performed, and termination of pregnancy (TOP) at 32 weeks.

The third case (our study), diagnosed at 32 weeks, carried a *de novo* frameshift *SCN2A* mutation (c.3043del, p.D1015Lfs*22). Its imaging features differed from previous cases: narrowing of the cavum septi pellucidi (CSP) on both ultrasound and MRI, plus cortical thickening and abnormal gyration on MRI, without ventriculomegaly. TOP was performed at 34 weeks due to fetal central nervous system abnormalities.

In conclusion, de novo *SCN2A* mutations are consistently associated with fetal central nervous system malformations and adverse pregnancy outcomes (epilepsy post live birth or TOP). However, imaging phenotypes exhibit heterogeneity—ventriculomegaly was the core feature in prior cases, while CSP narrowing and cortical thickening are the main characteristics of our case, providing additional clinical evidence for the prenatal diagnosis of such disorders.

## 4 Discussion

The pathogenic mechanisms of *SCN2A* variants leading to neurodevelopmental disorders remain incompletely understood, and the full disease spectrum is not yet fully delineated (Clatot et al., 2025). Our present study describes the first prenatal imaging phenotype associated with a frameshift *SCN2A* mutation, characterized by persistent cavum septi pellucidi (CSP) narrowing and progressive focal cortical thickening.

The cavum septi pellucidi (CSP), a key fetal brain structure whose absence or reduced size can indicate corpus callosum agenesis and is linked to schizophrenia spectrum disorders (Sichitiu et al., 2024; Shinar et al., 2020; Brisch et al., 2007), has not been reported in phenotypes associated with *SCN2A* mutations. By comparing the reported prenatal findings with postnatal brain malformations in previous SCN2A variant patients, we found that postnatal patients mainly presented with cortical dysplasia, predominantly polymicrogyria (PMG) in the frontoparietal, temporoparietoinsular and perisylvian regions, and some were complicated with midline structural anomalies such as corpus callosum hypoplasia (Clatot et al., 2025). Focal cortical thickening observed in our study may progress to cortical dysplasia after birth. While now only two cases of prenatal imaging manifestations associated with *SCN2A* gene mutations have been reported, which were mainly characterized by bilateral ventriculomegaly as described in Table 2 (Sauvestre et al., 2017; Bernardo et al., 2017). Ventriculomegaly represents the typical imaging feature of corpus callosum anomalies (Wu and Chen, 2024), and cavum septi pellucidi (CSP) narrowing serves as an early sign of midline structural anomalies that could be masked postnatally. Therefore, bilateral ventriculomegaly in fetuses reported previously and CSP narrowing reported in the present study may be the early intrauterine signs of postnatal corpus callosum malformations in *SCN2A*-mutant fetuses. To fully elucidate this dynamic evolutionary trajectory, larger sample sizes and long-term follow-up studies are essential.

*SCN2A* gene mutations underlie various infantile epilepsies (Howell et al., 2015), and a prenatal onset case has been documented (Sauvestre et al., 2017). Since the postnatal prognosis is generally poor in most cases, prenatal imaging plays a crucial role in early identification. A previously reported case of *SCN2A* missense variant -associated malformations of cortical development (MCD) showed diffuse cortical grey-white matter blurring and severe ventriculomegaly on fetal MRI (Sauvestre et al., 2017), whereas our case with a frameshift LoF variant presented with similar cortical blurring but a persistently narrow cavum septum pellucidum instead of ventriculomegaly, suggesting that different mutation types (missense vs. truncating) may correlate with distinct prenatal imaging trajectories.

Some studies showed that *SCN2A* loss-of-function variants typically cause intellectual disability and ASD with variable epilepsy, and gain-of-function variants are linked to early-onset epilepsies ranging from benign to severe, but many variants exhibit complex effects that defy simple functional classification, leaving the precise genotype–phenotype relationship unclear (Thompson et al., 2023; Berg et al., 2024; Meisler et al., 2021; Wolff et al., 2019). In this case, the observed truncation of Nav1.2 (p.D1015Lfs*22) disrupts critical channel domains (III–IV and C-terminal), consistent with a loss-of-function (LoF) mechanism. However, missense variants can also produce similar effects. For the latter, their impact cannot be predicted and must be assessed through functional studies, but the possibility of complete LOF cannot be ruled out *a priori*.

The mechanism by which *SCN2A* variants disrupt cortical development remains speculative. Studies have documented “paradoxical hyperexcitability” in the prefrontal or striatal neurons of adult mice deficient in *SCN2A*, indicating a potential connection to human *SCN2A*-related epilepsy (Spratt et al., 2021). However, these findings are inconsistent with other research outcomes (Miyamoto et al., 2019). Further research is needed to clarify the actual significance of neuronal “paradoxical hyperexcitability” for the pathological phenotypes in mice and patients with Nav1.2 deficiency.

This study has limitations. As a single-case report, the generalizability of the imaging phenotype remains uncertain. Larger cohorts are required to establish genotype–phenotype correlations and determine whether CSP narrowing is a consistent feature of *SCN2A* LoF variants. Additionally, the lack of postmortem histopathological validation limits our ability to confirm the cellular basis of imaging findings.

These findings translate into three actionable recommendations for prenatal clinical practice. First, the detection of unexplained cavum septi pellucidi (CSP) narrowing or focal cortical thickening should raise clinical suspicion for sodium channelopathies. Second, *SCN2A* should be included as a high-priority gene in diagnostic panels for fetal cortical malformations. Finally, with fetal MRI and exome sequencing becoming routine, the integration of dynamic imaging phenotypes with molecular data through multidisciplinary collaboration is imperative. This integrated approach will facilitate more accurate prognostication and inform personalized management strategies for families affected by these disorders.

## Data Availability

The datasets for this article are not publicly available due to concerns regarding participant/patient anonymity. Requests to access the datasets should be directed to the corresponding author.

## Footnotes

1
https://swissmodel.expasy.org/interactive

2
http://www.pymol.org
